# Influence of Aluminum Incorporation on the Adsorptive Performance of Silica-Based Supported Sulfonic Acid for the Chemical Recovery of Gaseous *O*-Xylene

**DOI:** 10.3390/molecules30051073

**Published:** 2025-02-26

**Authors:** Yaxu Wang, Jiaxuan Chai, Yining Li, Zichuan Ma

**Affiliations:** Hebei Key Laboratory of Inorganic Nano-Materials, College of Chemistry and Material Sciences, Hebei Normal University, Shijiazhuang 050024, China; yaxuwang@stu.hebtu.edu.cn (Y.W.); chaijx0123@163.com (J.C.); liyining0615@163.com (Y.L.)

**Keywords:** Al/Si ratios, CB-state, IP-state, *o*-xylene, adsorption

## Abstract

A group of silica-based supports with varying Al/Si ratios (S−x) was synthesized using the sol–gel method, followed by a chlorosulfonic acid modification to produce supported sulfonic acids (SA−x). The S−x and SA−x materials, along with their adsorption products, were characterized via techniques such as FTIR, BET, and HPLC-MS. The analysis revealed that the sulfonic acid groups in the SA−x materials existed in two anchoring states: the covalently bonded (CB) state [SiOx–O]^ɗ−^–SO_3_H^ɗ+^ and the ion-paired (IP) state AlO_y_^+^:OSO_3_H^−^. The sulfonation reactivity of the CB-state sulfonic acid was enhanced, whereas that of the IP-state counterpart was diminished. The incorporation of a minor quantity of aluminum ions (x = 0.1) markedly enhanced the adsorption efficiency of SAs for *o*-xylene, extending the reaction temperature range to 110–190 °C and increasing the breakthrough adsorption capacity (*Q*_B_) to 946.1 mg g^−1^. However, excessive aluminum ion incorporation was detrimental to the adsorption performance of SAs for *o*-xylene. SA−0.1 showed superior adsorptive capabilities and excellent recyclability, maintaining its performance over four consecutive adsorption/regeneration cycles with only a minor decrease of 4.5%. These findings suggest that SAs prepared with a minor amount of aluminum ions have significant potential for application as adsorbents for the removal of benzene series pollutants.

## 1. Introduction

Volatile organic compounds (VOCs), such as benzene, toluene, xylene isomers, acetone, dichloromethane, and others, are commonly encountered pollutants in both industrial and residential environments [1]. The presence of VOCs has been associated with adverse environmental impacts, including the formation of photochemical smog and haze, as well as significant health risks. These risks include respiratory diseases, sensory irritation, metabolic disorders, and, in extreme cases, carcinogenic effects [2,3]. Consequently, the emission levels of VOCs are regulated by stringent legal standards [4,5]. The removal and recovery of VOCs have garnered increasing attention and are considered a critical research challenge within the fields of separation and purification technology [6,7].

A variety of physico-chemical and biological technologies have been developed for the treatment of VOCs, including condensation, absorption, adsorption, incineration, catalytic combustion, biological degradation, and others [8,9,10,11,12,13]. Among these methods, adsorption stands out as a preferred technology for controlling VOC pollution due to its lower operational costs, process safety, and potential for adsorbent regeneration. Traditionally, temperature swing adsorption (TSA) and pressure swing adsorption (PSA) have been widely employed in industrial applications, and both techniques are capable of achieving adsorbent regeneration and adsorbate recovery [14,15,16]. However, in both of these processes, adsorption occurs via physical mechanisms at relatively low temperatures (for TSA) or at high pressures (for PSA), whereas desorption necessitates high temperatures or vacuum conditions. It is well documented that the textural properties of the adsorbent, such as specific surface area, pore volume, and pore width, have a substantial impact on its adsorption capacity. Nevertheless, these properties are prone to deteriorating over time due to pore collapse/clogging and surface contamination [17,18], which can hinder the widespread application of adsorbents. Consequently, it is imperative to develop more advanced adsorption technologies and materials for VOC decontamination [19].

A novel reactive adsorption methodology was proposed in our prior research for the removal and recovery of aromatic VOCs such as benzene, toluene, xylene, and ethylbenzene [20]. This method showed that silica-supported sulfuric acid (SSSA), prepared through sulfuric acid impregnation, serves as an effective adsorbent for removing *o*-xylene from gas streams. The mechanism involves the sulfonation of *o*-xylene on the SSSA surface. Unlike traditional TSA and PSA processes, this approach offers the distinct advantage of recovering the adsorbed aryl sulfonic acid and recycling the solid support, thereby demonstrating significant potential for the removal and recovery of benzene series compounds (BTEX) from waste gas streams. Recent studies have demonstrated that when sulfuric acid is employed as a precursor for the preparation of SSSA, the −SO_4_H groups are anchored on the silica surface in two distinct forms: a chemically bonded state (C-state) and a physisorbed state (P-state) [20,21,22]. Notably, the C-state sulfuric acid exhibits significantly higher reactivity towards *o*-xylene (a representative compound of BTEX) compared to its P-state counterpart. Furthermore, studies have established that by varying the silica structure [20], preparation method [21], and reaction medium [22], the amount or proportion of the C-state sulfuric acid on the SSSA material surface can be effectively regulated. These modifications essentially alter the hydroxyl density on the silica surface, thereby influencing the amount of the C-state sulfuric acid anchored via Si−O−S bonds. Given that element doping significantly influences the structure and surface hydroxyl groups of silica-based supports, it is of theoretical and practical significance to investigate the impact of doping on the reactivity of the C-state sulfuric/sulfonic acid sites in silica-based supports during the removal of BTEX pollutants via the reactive adsorption mechanism.

**Scheme 1:**
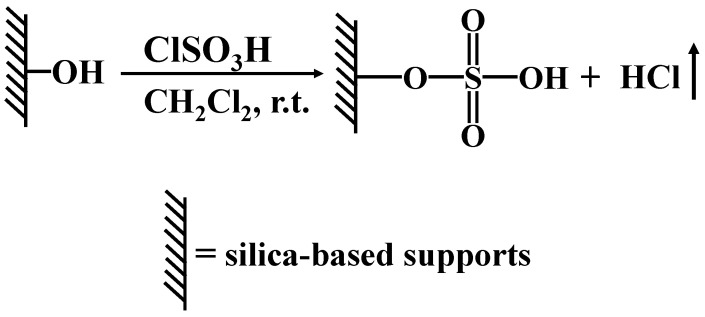
Preparation process for the SA−x series.

Therefore, silica-based supports with varying Al/Si ratios (designated as S−x, where x represents the Al/Si ratio in the support) were synthesized using the sol–gel method [23]. These supports were then reacted with chlorosulfonic acid in a dichloromethane medium to selectively produce the corresponding silica-supported sulfonic acids in their C-state form (SA−x) [24], as depicted in Scheme 1. The adsorptive performance of the synthesized SA−x materials for gaseous BTEX pollutants was evaluated using *o*-xylene as a representative compound. The influence of aluminum ion incorporation on the nature and reactivity of the anchored sulfonic acid sites with *o*-xylene was investigated. It was found that the sulfonic acid sites on the surface of the SA−x materials existed in two anchoring states: the covalently bonded state [SiOx–O]^ɗ−^–SO_3_H^ɗ+^ (CB state) and the ion-paired state AlO_y_^+^:OSO_3_H^−^ (IP state). The results indicated that the sulfonation reactivity of IP-state sulfonic acid with *o*-xylene was diminished, whereas the sulfonation reactivity of the CB-state sulfonic acid was enhanced. Consequently, the incorporation of aluminum primarily influenced the adsorption efficiency of SA−x towards *o*-xylene by altering the exposure levels of the CB- and IP-state sulfonic acids. Among the synthesized materials, SA−0.1 exhibited superior adsorptive capabilities for *o*-xylene and excellent recyclability, achieving a maximum *Q*_B_ of 946.1 mg g^−1^ and maintaining performance over four consecutive adsorption/regeneration cycles, with only a minor decrease of 4.5%. Therefore, it can be concluded that the silica-supported sulfonic acids prepared by incorporating a minor amount of aluminum ions have significant potential for application as adsorbents for the removal of BTEX pollutants.

## 2. Results and Discussion

### 2.1. Impact of Incorporation of Aluminum Ions on Reactivity of SA−x for O-Xylene Removal

**Figure 1:**
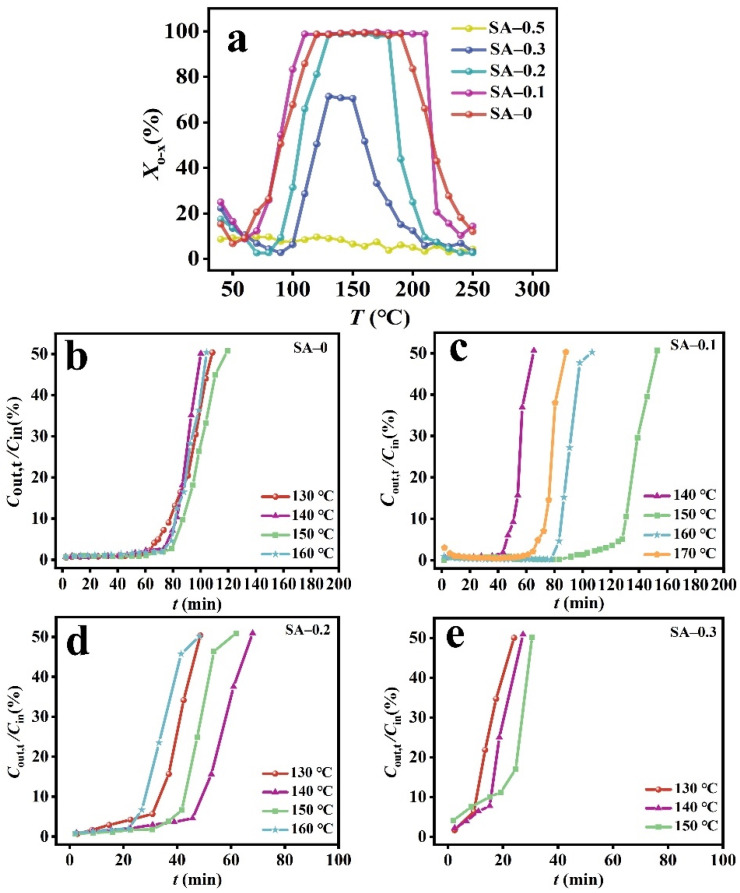
(**a**) Relationship between *o*-xylene *X*_o–x_ and *T* for the SA−x series. Adsorption breakthrough curves for *o*-xylene in (**b**) SA−0, (**c**) SA−0.1, (**d**) SA−0.2, and (**e**) SA−0.3 at different temperatures.

The reactivity of supported sulfuric/sulfonic acid materials with BTEX can be assessed through response curves that illustrate the variation in the removal rate as a function of temperature [20,21,22]. Figure 1a displays the relationship curves between *X*_o-x_ and *T* for various SA−x materials. Reactive regions with broad temperature ranges corresponding to *o*-xylene *X*_o-x_ ≥ 98.0% were observed for SA−0, SA−0.1, and SA−0.2, with the anterior (below the starting temperature) and posterior (above the terminal temperature) segments indicating the activation and deactivation effects, respectively, for the *o*-xylene removal [21]. Through further comparative analysis, it was determined that SA−0.1 exhibited the broadest reactive temperature region (110–210 °C) and the highest *X*_o-x_ (99.7%). SA−0 ranked second, with a reactive temperature region of 120 °C to 190 °C and *X*_o-x_ of 99.3%, while SA−0.2 placed third, demonstrating a reactive temperature region of 130 °C to 180 °C and *X*_o-x_ of 98.9%. When the Al/Si ratio was increased to 0.3, the reactive temperature region of SA−0.3 was narrowed to 130–150 °C, and its *X*_o-x_ value decreased to 70.5%. Notably, throughout the entire test temperature range, the removal rate of *o*-xylene by SA−0.5 consistently remained below 10%. These findings suggest that during the sol–gel preparation of silica supports, the incorporation of aluminum ions has a significant impact on the reactivity of supported sulfonic acid materials (SAs) towards *o*-xylene. Specifically, an optimal concentration of aluminum ions can effectively enhance the reactivity of SAs, whereas an excessive concentration can inhibit or even completely suppress their reactive performance.

Furthermore, isothermal reactive adsorption breakthrough curves were utilized to quantitatively assess the adsorptive performance of four reactive SA−x materials (excluding SA−0.5) for *o*-xylene. Figure 1b–e present the breakthrough curves for SA−0, SA−0.1, SA−0.2, and SA−0.3, respectively, at the specified temperatures. Table 1 presents the specific values of the *t*_B_ and *Q*_B_ metrics for each SA−x material, which were calculated based on the corresponding curves [20,25]. When SA−0, SA−0.1, and SA−0.2 were utilized as adsorbent bed materials for the removal of *o*-xylene, these materials exhibited prolonged periods of non-penetration. In contrast, SA−0.3 demonstrated a significantly shorter duration of effectiveness. The dynamic adsorption behavior of each material was influenced by the temperature to varying extents. Specifically, the reactive adsorption of SA−0.1 was the most sensitive to temperature changes, followed by that of SA−0.2, SA−0, and SA−0.3. The experimental results indicated that the optimal adsorption temperature for SA−0 and SA−0.1 was 150 °C, whereas the optimal temperature for SA−0.2 and SA−0.3 was 140 °C. Based on both the *t*_B_ and *Q*_B_ performance metrics under the optimal temperature conditions (Table 1), the reactivity of the SA−x samples towards *o*-xylene is in the order SA−0.1 > SA−0 > SA−0.2 > SA−0.3, which is consistent with the aforementioned conclusion.

### 2.2. Characterization of Structure and Surface Properties of SA−x Materials

**Figure 2:**
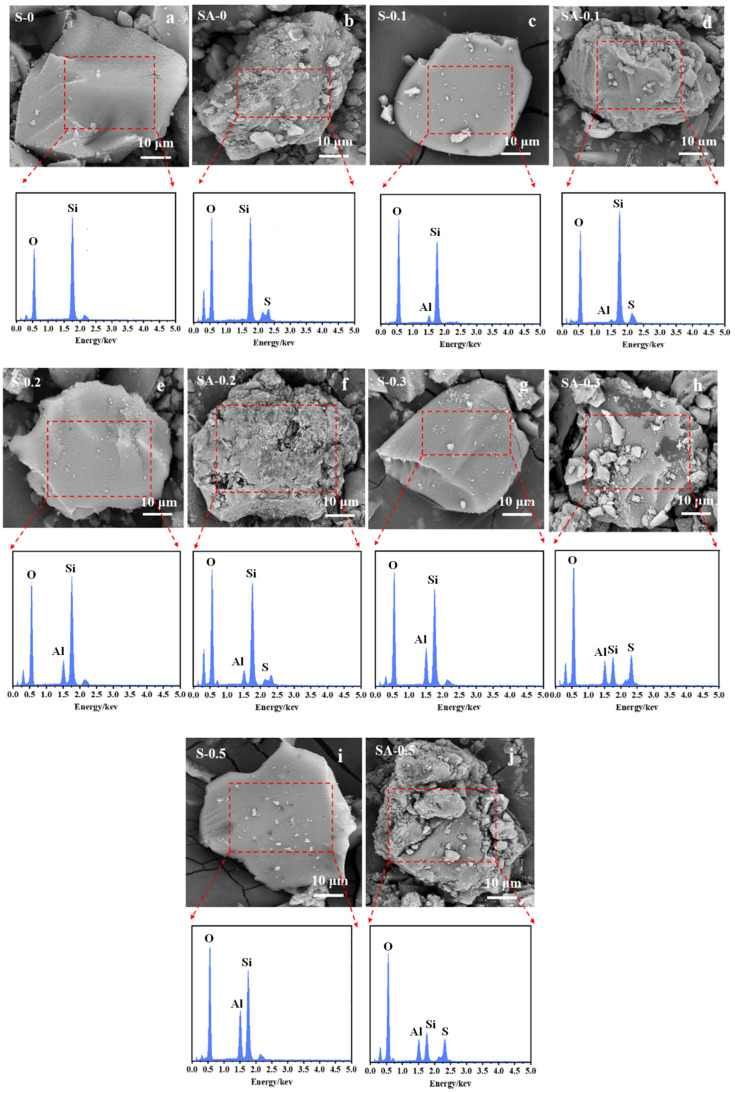
SEM images and corresponding EDS spectra of S−0 (**a**), SA−0 (**b**), S−0.1 (**c**), SA−0.1 (**d**), S−0.2 (**e**), SA−0.2 (**f**), S−0.3 (**g**), SA−0.3 (**h**), S−0.5 (**i**), and SA−0.5 (**j**).

Figure 2 concurrently shows the SEM images and corresponding EDS spectra of the S−x and SA− materials. Through comparative analysis, it is evident that chlorosulfonic acid modification (i.e., the introduction of sulfonic acid groups onto the S−x supports) induced significant morphological and compositional changes in the SA−x materials. The originally smooth surface of the monolithic gel fragments became notably rougher due to the etching effect of chlorosulfonic acid during the preparation process [26]. Furthermore, sulfur emerged as an external elemental component, with an atomic percentage ranging from 3.8% to 7.6% and a weight percentage between 6.2% and 12.0% (Table 2). These findings confirm the successful introduction of sulfonic acid groups onto the surface of all S−x materials [27]. By comparing the results of all tested materials, it was observed that the incorporation of aluminum ions had a negligible impact on the morphology of the silica-based supports. However, as the amount of incorporated aluminum increased (i.e., with higher Al/Si ratios), the aluminum content in the synthesized silica-based supports showed an upward trend, with the atomic percentage progressively increasing from 0% in the S−0 sample to 12.5% in the S−0.5 sample. Additionally, the aluminum content in the SA−x materials after sulfonic acid functionalization slightly decreased, likely due to the dilution effect caused by the introduction of sulfonic acid groups.

**Figure 3:**
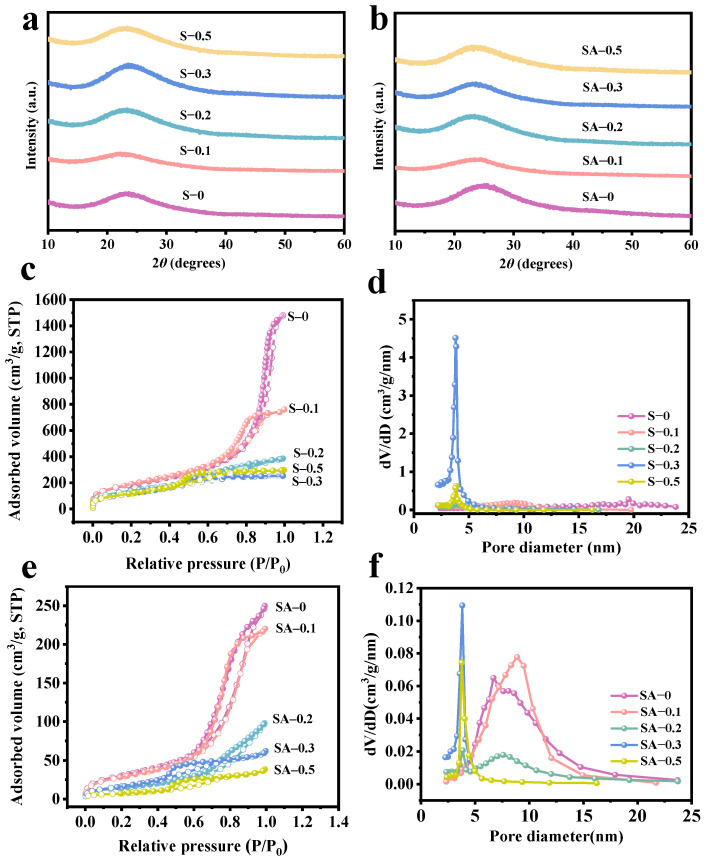
XRD patterns (**a**), N_2_ adsorption/desorption isotherms (**c**), and pore size distribution profiles (**d**) of the S-x supports; XRD patterns (**b**), N_2_ adsorption/desorption isotherms (**e**), and pore size distribution profiles (**f**) of the SA-x adsorbents.

Figure 3a,b illustrate the XRD patterns of the S−x supports and as-prepared SA−x materials, respectively. No distinct peaks corresponding to crystalline forms of SiO_2_ and Al_2_O_3_ were observed; instead, a broad diffraction band centered at approximately 2*θ* = 23° was evident, which could be attributed to the disordered agglomeration of amorphous silica particles of extremely small size during the sol–gel process [28]. This observation suggests that the incorporation of aluminum ions and the modification with chlorosulfonic acid did not significantly alter the bulk structure of the silica-based matrix. However, the incorporation of aluminum ions markedly altered the textural properties of the silica-based supports, including their specific surface area (*S*_BET_), total pore volume (*V*_T_), and average pore diameter (D_aver_) (Figure 3c,d and Table 3). Additionally, in conjunction with chlorosulfonic acid modification, it collectively influenced the textural characteristics of the prepared supported sulfonic acid materials (Figure 3e,f and Table 3). Specifically, among the five supports, S−0.1 had the largest *S*_BET_ and *V*_T_ values. With an increase in the Al/Si ratio, both *S*_BET_ and *V*_T_ showed a monotonically decreasing trend. Compared with that of S−0, the D_aver_ value of the aluminum-incorporated supports was significantly reduced. According to the preparation principle of the supported sulfonic acid materials, shown in Scheme 1, when these supports are used to prepare supported sulfonic acid materials, the sulfonic acid loading of the resulting SA−x depends on the number of hydroxyl groups on the support surface, and the number of hydroxyl groups is positively correlated with the specific surface area and pore volume of the support. Therefore, the sulfonic acid loading of SA−0.1 was the highest, reaching 8.1 mmol g^−1^ (Table 4). Compared with those of the corresponding S−x supports, the D_aver_ value of each SA−x was significantly increased, while the *S*_BET_ and *V*_T_ values were significantly reduced, which was mainly due to the strong polarity of the sulfonic acid group, resulting in the filling or blocking of the micro-/mesopores of the supports. The experimental results also showed that the *S*_BET_ and *V*_T_ values of SA−0 and SA−0.1 were similar, while the supported sulfonic acid materials with a higher Al/Si ratio showed worse textural properties. Therefore, these results initially explain the evolution trend of the reactivity of the SA−x materials in *o*-xylene removal and indicate that SA−0.1 has optimal reactive adsorption performance.

**Figure 4:**
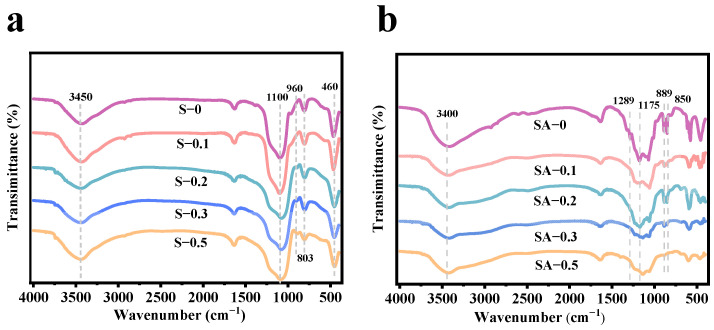
(**a**) FTIR spectra of the supports; (**b**) FTIR spectra of the samples.

FTIR spectra of the S−x supports are depicted in Figure 4a. These spectra exhibit a high degree of similarity, characterized by two broad and intense peaks centered at 3450 cm^−1^ and 1100 cm^−1^, a medium-intensity narrow peak at 460 cm^−1^, and two weak narrow peaks at 960 cm^−1^ and 803 cm^−1^. These features can be attributed to the superimposed absorption of O−H groups in Si−OH and H_2_O [29,30], the asymmetric stretching vibrations of the Si−O−Si and Si−O−Al backbones, the bending vibrations of Si−O bonds, the Si−OH vibration, and the symmetric stretching vibrations of Si−O−Si, respectively. This observation indicates that the incorporation of aluminum ions did not substantially alter the bulk and surface structures of the silica-based matrix, as further evidenced by the XRD analysis results. However, the broad band near 1100 cm^−1^ observed in the spectrum of the S−0 sample progressively shifted to 1075 cm^−1^ in the spectra of the S−0.3 and S−0.5 samples as the Al/Si ratio increased. Additionally, this band broadened, suggesting the formation of Si−O−Al bonds [31]. Compared to the FTIR spectra of these S−x supports, the spectra of the corresponding SA−x materials became more complicated (Figure 4b); they mainly exhibited additional bands at 1289, 1175, 889, and 850 cm^−1^, which were superimposed on the broad band associated with the asymmetric stretching vibrations of the Si−O−Si and Si−O−Al backbones in the supports. Due to the introduction of sulfonic acid groups, the original spectral band near 3450 cm^−1^ became broader and more intense, with a main shift towards lower wavenumbers (3400 cm^−1^). These characteristic changes provide compelling evidence that sulfonic acid groups were successfully anchored onto the S−x supports [20,32].

### 2.3. Analysis of Anchoring States of Sulfonic Acid Groups in the SA−x Materials

**Figure 5:**
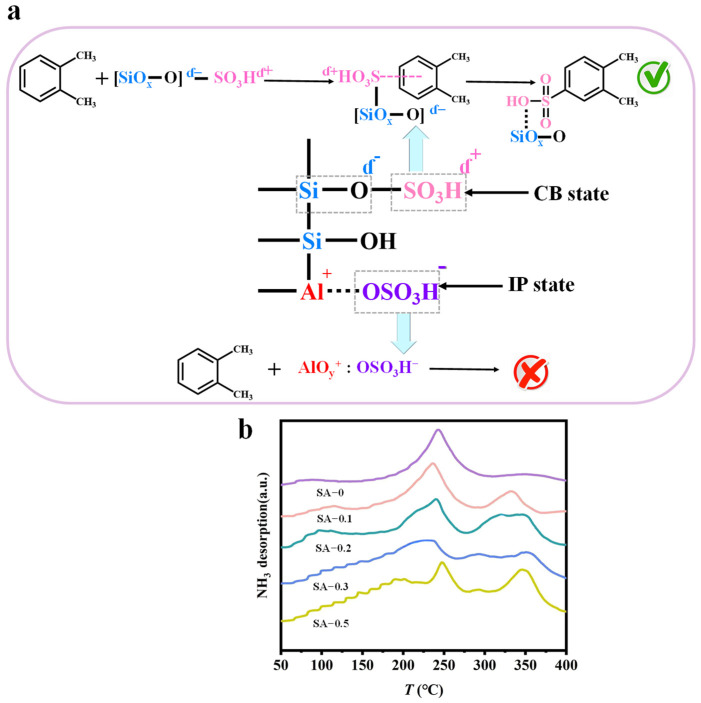
(**a**) Two anchoring states of the sulfonic acid and adsorption reaction equation of CB-state sulfonic acid with *o*-xylene; (**b**) NH_3_-TPD curves of SA−x materials.

As previously discussed, the SA−x materials exhibited varying sulfonic acid loadings, ranging from 6.2 to 8.1 mmol g^−1^ (Table 4), which can be attributed to differences in their textural properties and surface hydroxyl group densities. If we assume that the sulfonic acid groups anchored within the SA−x materials are in a uniform state and possess consistent *o*-xylene sulfonation reactivity, one would expect all tested SA−x samples to demonstrate comparably high reactivity and adsorptive performance. However, this assumption is inconsistent with the experimental findings presented in Section 2.1 of this study, particularly the observation that SA−0.5 exhibited no reactivity towards *o*-xylene. This discrepancy suggests that the sulfonic acid groups in different SA−x samples may exist in distinct anchoring states depending on their generation by Si−OH or Al−OH. In accordance with Scheme 1, when Si−OH reacts with chlorosulfonic acid, it is reasonable to infer that the resulting sulfonic acid groups are covalently bonded (CB) to the surface of the support ([SiOx–O]^ɗ−^–SO_3_H^ɗ+^). This occurs due to the comparable electronegativity between Si (1.90) and O (2.55), as well as between S (2.58) and O [33]. As illustrated in Figure 5a, the sulfonic acid sites in the CB state carry a positive charge, enabling them to undergo electrophilic substitution reactions, specifically sulfonation reactions, with *o*-xylene molecules [34,35]. In contrast, given the significantly lower electronegativity of Al (1.61) compared to Si [33], the hydroxyl groups (Al−OH) associated with Al result in the sulfonic acid groups being anchored on the support surface via ion-paired (IP) bonding. This interaction transforms the sulfonic acid groups into negatively charged hydrogen sulfate groups (AlO_y_^+^:OSO_3_H^−^), thereby inhibiting their participation in the electrophilic sulfonation reaction with *o*-xylene molecules (see Figure 5a).

The coexistence of the CB- and IP-state sulfonic acid groups in the aluminum-incorporated SA-x materials was further confirmed by means of NH_3_-TPD characterization. As illustrated in Figure 5b, from 50 °C to 400 °C, the curve of SA−0 showed only one broad peak centered at approximately 242 °C, while the curves of the other four aluminum-containing samples all exhibited two broad peaks, with their center positions located at approximately 236–247 °C and 332–348 °C. Prior to the temperature-programmed desorption treatment, NH_3_ molecules form hydrogen bonds with the sulfonic acid groups on SA−x materials and subsequently adsorb onto their surfaces from the gas phase. Since the acidity of AlO_y_^+^:OSO_3_H^−^ is greater than that of [SiOx–O]^ɗ−^–SO_3_H^ɗ+^, it can be expected that the hydrogen bond of NH_3_ with the IP-state sites is stronger than that with the CB-state sites. Apparently, the desorption peak located in the low-temperature region is attributed to sulfonic acid groups in the CB state derived from Si–OH, while the other desorption peak located in the higher-temperature region is attributed to sulfonic acid groups in the IP state derived from Al–OH.

In summary, the incorporation of aluminum ions leads to the formation of IP-state sulfonic acid sites and alters the textural properties of silica-based supports, which, in turn, influences the generation of CB-state sulfonic acid sites. Consequently, the incorporation of a small amount of aluminum ions (at an Al/Si ratio of 0.1) significantly enhances the reactivity and adsorption performance of SA−0.1 towards *o*-xylene. This enhancement is attributed to the increased specific surface area, which exposes more Si–OH groups, thereby promoting the formation of CB-state sulfonic acid groups. However, as the Al/Si ratio increases further, the specific surface area of the support gradually decreases. This reduction results in an increase in the number of IP-state sulfonic acid sites associated with Al–OH and a decrease in the number of CB-state sulfonic acid sites, leading to diminished reactivity and adsorption capacity of SA−x towards *o*-xylene. When the Al/Si ratio reaches 0.5, the high concentration of IP-state sulfonic acid sites severely hinders the electrophilic reaction between CB-state sulfonic acid sites and *o*-xylene molecules, primarily due to electrostatic interactions.

### 2.4. Characterization of the Adsorbed Product and Evaluation of the Reusability of the Spent Material

**Figure 6:**
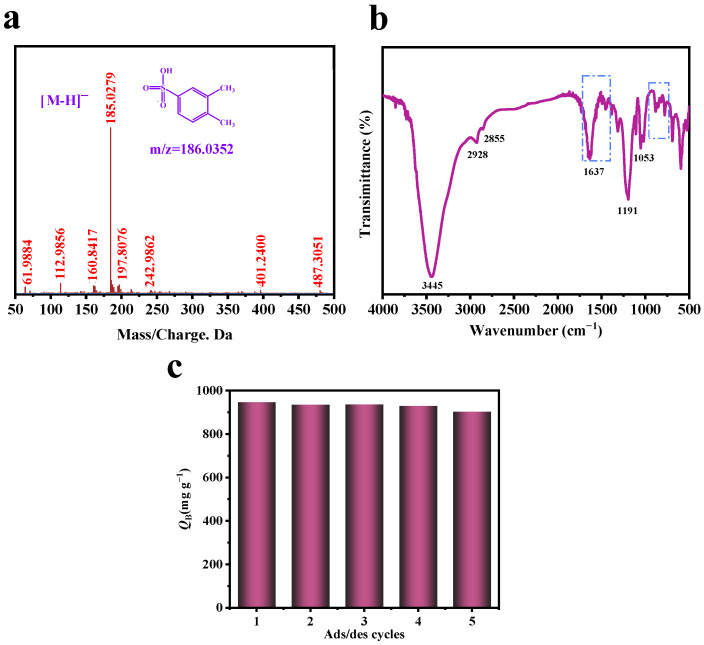
(**a**) ESI-MS spectrum of adsorption product; (**b**) FTIR spectrum of adsorption product; and (**c**) reusability of SA−0.1 in repeated adsorption/desorption cycles.

Methanol solvent was utilized to elute the adsorbed product from spent SA−0.1 material and the extracted product was subsequently characterized using HPLC-MS and FTIR spectroscopy. In the negative ion mode, as shown in Figure 6a, the mass spectrum exhibited a prominent molecular ion peak at *m*/*z* = 185 for the extracted product, indicating that the adsorption product had a relative molecular mass of 186. According to Figure 6b, the absorption band at ~3440 cm^−1^ corresponded to the –OH stretching vibration of the –SO_3_H group [36]. The peaks at 2928 cm^−1^ and 2855 cm^−1^ were attributed to the C–H stretching vibrations of the aryl–CH_3_ group [37], while the four bands in the range of 1637–1383 cm^−1^ were assigned to in-plane skeletal vibrations of the benzene ring. The bands at 1053, 1023, and 786 cm^−1^ were characteristic of out-of-plane bending vibrations of the –CH groups of the benzene ring [38]. Additionally, the bands at 1315 and 1191 cm^−1^, corresponding to the stretching vibration of O=S=O, confirmed the presence of sulfonated *o*-xylene [39]. Further evidence of sulfonation was provided by the peaks at 1108 and 827 cm^−1^, which corresponded to the stretching vibration of the C–S bond [40]. Based on these findings and those of our previous studies [20,21,41], it was concluded that 3,4-dimethylbenzenesulfonic acid was produced in the reactive adsorption process of *o*-xylene with the SA−0.1 material (see Figure 5a).

Figure 6c illustrates the results of the adsorption/desorption/re-modification cycles for *o*-xylene removal using the SA−0.1 material. Following the recovery of 3,4-dimethylbenzenesulfonic acid from spent SA−0.1 via methanol extraction, the SA−0.1 support was regenerated. By reapplying the chlorosulfonic acid modification, a new SA−0.1 material was prepared and utilized for subsequent reactive adsorption of *o*-xylene. After four cycles of adsorption, extraction, and re-modification, the *Q*_B_ values of each regenerated SA−0.1 material exhibited only minor variations compared to the initial performance, ranging from 903.2 mg g^−1^ to 946.1 mg g^−1^, thereby demonstrating the exceptional reusability of the SA−0.1 material.

## 3. Materials and Methods

### 3.1. Reagents and Materials

The following chemicals were obtained from YongDa Chemical Reagent (Tianjin, China): aluminum nitrate nonahydrate (Al(NO_3_)_3_·9H_2_O, 98%), dichloromethane (CH_2_Cl_2_, 98%), methanol (CH_4_O, 98%), ethanol (C_2_H_6_O, 99.7%), ammonium hydroxide (NH_3_·H_2_O, 25–28%), hydrochloric acid (HCl, 36–38%), sodium hydroxide (NaOH, ≥96%), potassium dihydrogen phosphate (KH_2_PO_4_, 99.5%), and ethylene glycol (C_2_H_6_O_2_, 98%). Tetraethyl orthosilicate (TEOS, 98%) was purchased from Shanghai Macklin Biochemical (Shanghai, China). Chlorosulfonic acid (ClSO_3_H, ≥98%) was obtained from AiKeDa Chemical Reagent (Chengdu, China). *O*-xylene (C_8_H_10_, 99%) was purchased from Kemio Chemical Reagent (Tianjin, China). All the above reagents were used as purchased, without any purification or additional processing.

### 3.2. Synthesis of Silica-Based Supports with Different Al/Si Ratios and Their Supported Sulfonic Acids

A series of silica-based supports with varying Al/Si ratios (S−x, x = 0, 0.1, 0.2, 0.3, or 0.5) were synthesized via the sol–gel method [23], utilizing TEOS as the silicon precursor and Al(NO_3_)_3_·9H_2_O as the aluminum dopant. As a typical example, the preparation procedure of S−0.1 was as follows: Quantities of 0.01 mol of TEOS and 0.1 mol of Al(NO_3_)_3_·9H_2_O were dissolved in 60 mL of anhydrous ethanol. The solution was subsequently stirred and refluxed in a water bath at 80 °C for 1 h to achieve an Al/Si mole ratio of 0.1. Thereafter, the pH of the system was adjusted to 7.0 by adding a 10 (wt.)% ammonium hydroxide solution, during which a white gel gradually formed. The mixture was further aged under the same conditions for 24 h, followed by drying in an oven at 80 °C for 12 h. Subsequently, the sample was transferred to a muffle furnace and calcined in an air atmosphere at 500 °C for 3 h. After cooling to room temperature, the S−0.1 sample was sieved to ensure a particle size of less than 0.15 mm and subsequently stored in a desiccator for future use. The remaining samples were prepared by following identical procedures and denoted as S−0, S−0.2, S−0.3, or S−0.5, according to the Al/Si ratio.

In the preparation of supported sulfonic acid materials [24], 3 g of the S−x sample was dispersed in 15 mL of dichloromethane and stirred for 30 min to form suspension A. Separately, 2.5 mL of chlorosulfonic acid was dissolved in 10 mL of dichloromethane to prepare solution B. Under continuous stirring, solution B was then gradually added dropwise to suspension A, followed by prolonged stirring for an additional 3 h. The resulting mixture was filtered and washed three times with 10 mL portions of dichloromethane. The supported sulfonic acid materials were subsequently dried in a vacuum oven at 60 °C for 12 h. After cooling to ambient temperature, the particle size of the materials was adjusted to between 0.15 mm and 0.28 mm through processes such as tableting, grinding, and sieving. The final products were stored in a desiccator for future use. These materials, corresponding to their respective supports, were designated as SA−0, SA−0.1, SA−0.2, SA−0.3, and SA−0.5. The loading of sulfuric acid in these materials was quantified using an acid–base titration technique.

### 3.3. Characterization

The morphology and chemical composition of the materials were investigated using an S-4800 cold-field emission scanning electron microscope equipped with an INCA 350 energy-dispersive spectrometer accessory (SEM-EDS; Hitachi, Chiyoda City, Japan). Fourier-transform infrared spectra (FTIR; KBr pellets) were recorded on a TENSOR 27 spectrometer (Bruker AXS, Karlsruhe, Germany). The N_2_ adsorption/desorption isotherms of the materials were measured at 77 K using a Kubo X1000 automatic surface area and pore analyzer (Beijing Builder Co., Ltd., Beijing, China). Prior to testing, the materials were subjected to treatment at 100 °C for 1.5 h in an MD-300 preprocessor (Beijing Builder Co., Ltd., Beijing, China). The specific surface area, pore volume, pore width, and pore size distribution were determined using the Brunauer–Emmett–Teller (BET) and Barrett–Joyner–Halenda (BJH) models. The X-ray diffraction (XRD) analysis of the adsorbent was carried out using a D8 Advance X-ray diffractometer operating with Cu Kα radiation (λ = 0.154 nm; Bruker AXS, Karlsruhe, Germany). The acid sites of samples were studied via NH_3_ temperature-programmed desorption (NH_3_-TPD) experiments in a PCA-1200 chemisorption analyzer (Beijing Builder Co., Ltd., Beijing, China). In order to remove surface contaminants, 50 mg of sample (0.15−0.28 mm) was pretreated in flowing Ar at 200 °C for 2 h, then cooled down to room temperature. At this temperature, the flow was switched to He (99.999%), and then NH_3_ was supplied to the samples for 1 h; the NH_3_ was desorbed at a heating rate of 10 °C min^−1^ from room temperature to 400 °C. The adsorbed *o*-xylene derivative was subjected to analysis using high-performance liquid chromatography–mass spectrometry (HPLC-MS) on an Agilent 1260 system (Agilent Technologies, Santa Clara, CA, USA).

### 3.4. Dynamic O-Xylene Adsorption Experiments

Common dynamic adsorption experiments were developed to evaluate the performance of each SA−x material in removing *o*-xylene from gas streams; these experiments were performed on self-constructed equipment with a fixed-bed column. In each independent experiment, high-purity nitrogen was utilized as the carrier gas, with its flow rate (V_g_) regulated by a mass flow controller to maintain a constant value of 0.050 L min^−1^. A stream was generated by bubbling nitrogen into a gas wash bottle containing liquid *o*-xylene, which was maintained at 0 °C using a low-temperature coolant circulation pump (with ethylene glycol serving as the coolant), resulting in an inlet *o*-xylene concentration (*C*_in_) of 7.5 mg L^−1^. Then, the simulated gas was passed through a quartz tube (6 mm i.d. × 40 cm) packed with SA−x adsorbent (50 mg, 0.15–0.28 mm) blended with inert quartz sand (100 mg, 0.180–0.425 mm), and the outlet concentration (*C*_out_) of *o*-xylene was monitored using the GC7900 gas chromatograph (Tianmei, Shanghai, China) equipped with a flame ionization detector FID and a polyethylene glycol (PEG)-20 M capillary column (30 m × 0.32 mm i.d. × 0.5 µm df). The temperature range for the reaction between the SA−x adsorbent and *o*-xylene was determined using a temperature-programmed process from room temperature to 250 °C at a rate of 2 °C min^−1^. The impact of the adsorption temperature on the adsorption capacity of the samples for *o*-xylene was investigated by constructing breakthrough curves. The removal ratio of *o*-xylene (*X*_o-x_, %) and the breakthrough adsorption capacity (*Q*_B_, mg g^−1^) were calculated using Equations (1) and (2), respectively.

(1) $$X_{o-x}=\frac{{C_{\mathrm{in}}-C}_{\mathrm{out},t}}{C_{\mathrm{in}}}\times 100\%$$

(2) $$Q_{B}=\frac{V_{g}C_{\mathrm{in}}}{m}\int_{0}^{t_{B}}\left(1-\frac{C_{\mathrm{out},t}}{C_{\mathrm{in}}}\right)dt$$

Here, *m* (g) is the adsorbent mass; *t*_B_ (min) denotes the breakthrough time, which is the time taken for the *C*_out_/*C*_in_ ratio to reach 0.05; and the remaining symbols are as previously defined.

## 4. Conclusions

Five silica-based precursors with varying Al/Si ratios (designated as S−x, where x = 0, 0.1, 0.2, 0.3, or 0.5) were synthesized via the sol–gel method. Subsequently, corresponding supported sulfonic acid materials (SA−x) were prepared using these precursors as supports through chlorosulfonic acid modification. NH_3_-TPD characterization and theoretical analyses indicated that the sulfonic acid groups in the SA−x materials exist primarily in two states: sulfonic acid groups in the CB state exhibit strong reactivity and adsorption performance towards *o*-xylene, whereas those in the IP state do not possess these properties. The incorporation of a small amount of aluminum ions significantly enhanced the reactivity and adsorption performance of SA−0.1 towards *o*-xylene, achieving a maximum adsorption capacity (*Q*_B_) of 946.1 mg g^−1^ and excellent recyclability. This enhancement was attributed to an increased specific surface area, which exposed more Si–OH groups, thereby promoting the formation of CB-state sulfonic acid groups. As the Al/Si ratio increased further, the number of sulfonic acid groups in the IP state associated with Al–OH increased, while the number in the CB state decreased, leading to diminished reactivity and adsorption capacity of SA−x towards *o*-xylene. Therefore, this study suggests that silica-supported sulfonic acids prepared by incorporating a minor amount of aluminum ions have significant potential for application as adsorbents for the removal of BTEX pollutants.

## Figures and Tables

**Table 1:** Adsorption parameters of SA-x materials for *o*-xylene at different temperatures.

*T* (°C)	SA−0		*T* (°C)	SA−0.1		*T* (°C)	SA−0.2		*T* (°C)	SA−0.3	
	*t*_B_ (min)	*Q*_B_ (mg g^−1^)		*t*_B_ (min)	*Q*_B_ (mg g^−1^)		*t*_B_ (min)	*Q*_B_ (mg g^−1^)		*t*_B_ (min)	*Q*_B_ (mg g^−1^)
130	70.2	520.0	140	46.4	351.6	130	27.4	199.8	130	19.3	58.0
140	77.3	571.5	150	127.5	946.1	140	45.9	342.5	140	22.2	63.9
150	81.2	598.7	160	83.5	623.2	150	39.1	287.0	150	8.0	27.4
160	79.1	584.1	170	68.4	510.2	160	24.7	183.5	—	—	—

**Table 2:** Surface element contents of the supports and samples based on the EDS measurements.

Support	Element	Atom. (%)	wt. (%)	Sample	Element	Atom. (%)	wt. (%)
S−0	O	65.6	52.1	SA−0	O	68.9	55.1
	Si	34.4	47.9		Si	24.0	3.6
	−	−	−		S	7.1	11.3
S−0.1	O	72.3	59.9	SA−0.1	O	66.5	52.4
	Al	2.5	3.5		Al	2.2	2.9
	Si	25.2	36.6		Si	23.7	32.7
	−	−	−		S	7.6	12.0
S−0.2	O	64.9	51.5	SA−0.2	O	67.3	53.4
	Al	5.5	7.4		Al	3.2	4.3
	Si	29.6	41.1		Si	23.3	32.4
	−	−	−		S	6.2	9.9
S−0.3	O	65.4	52.2	SA−0.3	O	67.1	53.4
	Al	7.5	10.1		Al	69.9	9.3
	Si	27.1	37.7		Si	20.3	28.3
	−	−	−		S	5.7	9.0
S−0.5	O	63.5	50.2	SA−0.5	O	62.8	48.9
	Al	12.5	16.6		Al	10.8	14.2
	Si	24.0	33.2		Si	22.6	30.9
	−	−	−		S	3.8	6.0

**Table 3:** Textural parameters of the S−x supports and SA−x adsorbents.

Support	*S*_BET_ (m^2^ g^−1^)	*V*_T_ (cm^3^ g^−1^)	*D*_aver_ (nm)	Adsorbent	*S*_BET_ (m^2^ g^−1^)	*V*_T_ (cm^3^ g^−1^)	*D*_aver_ (nm)
S−0	672	2.29	6.82	SA−0	113	0.39	9.36
S−0.1	695	1.17	3.38	SA−0.1	106	0.36	6.42
S−0.2	510	0.59	2.34	SA−0.2	55	0.15	5.49
S−0.3	466	0.39	1.76	SA−0.3	63	0.096	3.05
S−0.5	361	0.46	2.22	SA−0.5	29	0.059	4.26

**Table 4:** Loading of sulfonic acid in SA−x materials.

Sample	SA−0	SA−0.1	SA−0.2	SA−0.3	SA−0.5
Loading of sulfonic acid (mmol g^−1^)	7.8	8.1	7.2	6.6	6.2

## Data Availability

The data are contained within the article. The data presented in this study are available upon request.
